# Exoskeletons for Mobility after Spinal Cord Injury: A Personalized Embodied Approach

**DOI:** 10.3390/jpm12030380

**Published:** 2022-03-01

**Authors:** Giuseppe Forte, Erik Leemhuis, Francesca Favieri, Maria Casagrande, Anna Maria Giannini, Luigi De Gennaro, Mariella Pazzaglia

**Affiliations:** 1 Dipartimento di Psicologia, “Sapienza” Università di Roma, Via dei Marsi 78, 00185 Rome, Italy; erik.leemhuis@uniroma1.it (E.L.); annamaria.giannini@uniroma1.it (A.M.G.); luigi.degennaro@uniroma1.it (L.D.G.); mariella.pazzaglia@uniroma1.it (M.P.); 2 Body and Action Lab, IRCCS Fondazione Santa Lucia, Via Ardeatina 306, 00179 Rome, Italy; 3 Dipartimento di Psicologia Dinamica, Clinica e Salute, Università di Rome “Sapienza”, Via Degli Apuli 1, 00185 Rome, Italy; maria.casagrande@uniroma1.it

**Keywords:** spinal cord injuries, exoskeleton, interoception, embodiment, pain, body image, body representation, taVNS, cardiovascular

## Abstract

Endowed with inherent flexibility, wearable robotic technologies are powerful devices that are known to extend bodily functionality to assist people with spinal cord injuries (SCIs). However, rather than considering the specific psychological and other physiological needs of their users, these devices are specifically designed to compensate for motor impairment. This could partially explain why they still cannot be adopted as an everyday solution, as only a small number of patients use lower-limb exoskeletons. It remains uncertain how these devices can be appropriately embedded in mental representations of the body. From this perspective, we aimed to highlight the homeostatic role of autonomic and interoceptive signals and their possible integration in a personalized experience of exoskeleton overground walking. To ensure personalized user-centered robotic technologies, optimal robotic devices should be designed and adjusted according to the patient’s condition. We discuss how embodied approaches could emerge as a means of overcoming the hesitancy toward wearable robots.

## 1. Introduction

Spinal cord injuries (SCIs) are devastating life events that bring abrupt and disruptive changes into a person’s life and entail several secondary health challenges [1]. According to estimations, around 180,000 people worldwide are completely or partially paralyzed and have limited sensation due to SCIs [2,3]. The number of SCIs has increased in recent decades, primarily in individuals under the age of 30 [1]. The magnitude of functional recovery can vary considerably between individuals, depending on the location and severity of the spinal cord lesion. Damage to the spinal cord causes a brain–body disconnection of the body parts below the spinal cord lesion, with the interruption of efferent (motor) and afferent (sensory) information. The level of injury (indicated by the name and number of vertebrae involved) defines the portion of the body affected by the disconnection. The lesion’s severity (or completeness) determines the amount and quality of residual sensory and motor abilities.

This leads to a wide variability in clinical conditions: from complete numbness and inability to move to complete sensation in the absence of motor ability or intermediate situations with residual, but not complete, sensation and movement [4]. Symptoms typical of neurological lesions leading to disconnection of the body from the central nervous system may further manifest as chronic painful and nonpainful sensations (i.e., phantom sensation or neuropathic pain) of different etiologies, further complicating the complex constellation of symptoms in these patients.

Currently, there is no absolute cure, and recovering the mobility of paralyzed limbs is a high priority [5]. In this context, manual wheelchairs are the primary devices used for moving an immobile body. However, patients with SCIs commonly experience several limitations in the form of secondary medical issues concerning body paralysis and immobility, such as pain, muscle atrophy, obesity, coronary heart disease, and diabetes. Thus, the focus on assistive technologies for ambulating SCIs has increased over the past few years, mainly on wearable robotic legs.

Exoskeletons denote one such example of assistive technologies providing powered hip and knee motion to help individuals with SCIs initiate and control basic locomotion movements (for example, standing upright, walking, turning, climbing, and descending stairs) [6]. A wide range of lower-limb exoskeletons is under development. Several are available for research purposes, and some have already met the requirements to be officially recognized as rehabilitative and assistive devices (for a review, see Reference [7]).

The potential of the exoskeleton for gait recovery in SCIs has been demonstrated by objective improvements in performance, such as balance, walking distance, velocity, and duration [8,9]. In addition, a case report showed that two non-ambulatory patients with incomplete spinal lesions were able to walk again after exoskeleton training [10]. Standing and walking in an upright position through the use of an exoskeleton may also help prevent secondary health complications after SCIs, such as spasticity, impaired cardiovascular function, and muscle tone [11,12], promoting physical health and well-being. Surprisingly, despite the physical compensation of motor loss, the medical and societal pressure to walk, and great technological advances, only a small number of patients choose to use an exoskeleton [13]. However, the difficulty with exoskeletons for individuals with SCIs is that their utilization is very similar to dancing with a bad partner to facilitate a movement that the body is incapable of achieving. The notion of embodiment establishes how the brain can appropriately control these devices as one’s real body part.

With this perspective study, we aimed to highlight the homeostatic role of autonomic and interoceptive signals and their possible integration into a personalized experience of exoskeleton overground walking. To this end, we first elucidated high-quality technology available for SCIs for identifying the current gaps and potential benefits for patients. We considered aspects that may play a critical role in better adapting the body of a person with a SCI to the use of EXO to provide a new perspective on the personalized use of exoskeletons. Modulation of the homeostatic and autonomic signals during exoskeleton use could improve the interaction between SCI patients and the technological device, increasing the stability of the system and maximizing movement outcomes with exoskeletons into a single natural (joint) action.

## 2. Which Exoskeleton?

Despite the presence of several EXOs on the market currently, there are only three exoskeletons that have been approved by the U.S. Food and Drug Administration: ReWalk, Ekso, and Indego (see Table 1). All systems are class 2 medical devices tested in different environments and with specific inclusion and exclusion criteria for SCIs. ReWalk and Indego are approved for community and institutional use, while Ekso is approved only for use in a medical facility under trained medical supervision. Several studies have been conducted to investigate the safety and feasibility of the ReWalk [9,14,15,16], Ekso [5,17], and Indego systems [18,19]. In addition, some reviews have compared different exoskeleton systems as assistive tools for rehabilitation in chronic SCI populations [13,14,15,16,17,18,19].

Several parameters were considered when analyzing the feasibility of exoskeleton use, including the time and frequency of sessions, steps taken, distance traveled, and change in overall competence with use. In addition, several typical functional outcomes, such as the Spinal Cord Independence Measure and Functional Independence Measure (FIM), were evaluated. Considering the results of these reviews, only one study found no differences between assisted walking with the exoskeleton and traditional therapeutic approaches such as electrical stimulation, stretching, and strength training [16]. However, a limitation of the study could be the consideration of only an early generation of exoskeletons before the current advanced progress.

A meta-analysis by Miller et al. (2016) examined the safety and efficacy of these devices [12]. It included 14 studies (eight ReWalk, three Ekso, two Indego, and one unspecified exoskeleton) with a total of 111 patients and concluded that exoskeletons are safe to use and can provide significant health benefits. These findings were confirmed by a more recent meta-analysis that found greater benefits in rehabilitation and reintegration [7]. While individuals with SCIs consider exoskeletons a positive and desirable innovation, their use as both therapeutic and mobility devices at home is still limited [4]. To date, very few studies have reported contrasting attendance rates whenever a training program was offered [6,20]. Data on the evaluation of compliance with ambulation sessions are scarce. A few studies reported cases that dropped out during training due to discomfort, injuries, or adverse events during training [6,21,22]. Clearly, more studies are required.

## 3. The Exoskeleton and the Body: Bodily (and More) Effects of ReWalk

Among individuals with SCIs, treating secondary conditions after a SCI is a high priority [8,9]. The benefits of walking with an exoskeleton include strengthening impaired muscles, walking speed and efficiency, and secondary conditions after a SCI, such as spasticity [9,23], bone density, lean body mass, muscle tone, pain, and changes in cardiovascular and bladder and bowel functions. Improvements in mood and mental state and overall impact on quality of life have also been reported.

### 3.1. Spasticity

Spasticity, defined as an increase in muscle tone and tendon reflexes, is a common symptom of SCIs. Spasticity is reported in 66–78% of patients with SCIs [24]. More evidence suggests a significant improvement in spasticity scores on the modified Ashworth scale directly after an exoskeleton session [9,23] in all muscle groups [11] but not in all patients [25]. In contrast, Kressler et al. (2014) reported no change in spasticity with exoskeleton-assisted walking [26]. Despite the small sample sizes in the studies and the tendency to include self-reported data, the evidence of an effect on spasticity following exoskeleton training was reported. However, the effect could not be noticed four weeks after the training program ended [11] and may be predicated on initial low and high spasticity levels [10].

### 3.2. Musculoskeletal

Prolonged wheelchair use is known to lead to decreased bone mineral density, leading to an increased risk of fractures (approximately 25–46%) [27], muscle mass atrophy, and increased fat mass [28], increasing the risk of weight gain and metabolic complications [29]. Despite the published studies with small samples, different studies have noted musculoskeletal improvements after a 6-week exoskeleton intervention [28,29,30]. Exoskeletons are relevant in maintaining the body composition, increasing bone mineral density, and contributing to lean body mass.

### 3.3. Pain

SCIs include the development of chronic and neuropathic pain (from 26% to 96% individuals) [31] that is notoriously difficult to treat [32,33]. Although various treatments are available (such as drugs and physical and psychological therapies), pain is refractory in a significant proportion of people with SCIs (from 5 to 37%) [31]. The need for better access to effective noninvasive treatment options to alleviate pain makes the effect of powered exoskeletons on pain of particular interest [9,10,23,25,26,34,35,36].

Exoskeleton adoption reduces the intensity of neuropathic pain [26,37] and nociceptive pain [11,23]. Although pain improvement has been reported after each training session [35], as well as postintervention [38], in patients with SCIs monitored after 60 training sessions, as well as a one-year follow-up, no recurrence of pain was observed [37]. However, short-term alterations in the type, intensity, and location of pain between training sessions were not always observed [11,25], thus indicating that a longer duration of training is required for exoskeleton training in reducing neuropathic pain. However, the risk of developing painful pressure points (i.e., mild upper extremity pain/soreness and lower back pain) caused by balancing requires upper body strength and crutches when using an exoskeleton [11,39]. In summary, even in the case of pain, a potential positive effect of exoskeleton-assisted walking on neuropathic pain was found, although it could be a potential pain generator when called to move the entire body weight.

### 3.4. Cardiovascular Health

An increased and accelerated risk of cardiovascular disease has been observed in people with SCIs [40,41]. Physical activity and exercise are protective factors for cardiovascular diseases. In this sense, exoskeletons may provide a viable alternative for people with SCIs who have limited physical activity. Some studies have shown that exoskeleton-assisted walking increases the heart rate and may positively impact cardiovascular health in chronic patients with SCIs [42,43]. However, when trying to quantify the required effort in terms of energy expenditure, the conclusions are not univocal. If some studies have evaluated exoskeleton-assisted overground walking as equivalent to a moderate exercise activity, it would not be so in other cases where the energy expenditure would not exceed that of a normal walk [26,34]. In summary, exoskeleton walking may be an option for regular exercise with a positive impact on cardiorespiratory health, but this is based only on cohort studies with small sample sizes.

### 3.5. Neurogenic Bowel

Neurogenic bowel is another common secondary medical complication following SCIs [44]. Based on previous studies examining the benefits of upright posture and mobility on bowel motility, it was hypothesized that powered exoskeletons could improve bowel function [9,11,25,34,35]. Huang et al. (2015) showed that 20 min of exoskeleton use significantly reduced both enema dose and defecation time [45]. In addition, other studies have reported subjective improved bowel regularity [9,34]. Overall, the studies suggest a potential benefit for bowel function, although a study of six individuals with SCIs did not report an improvement of bowel function [35].

### 3.6. Physical Functioning and Physical Well-Being

Walking is a top priority in patients with SCIs [46]. A wearable exoskeleton to assist walking provides a realistic possibility of fulfilling this priority, even in patients with complete or incomplete paralysis. Therefore, the psychological impact of rehabilitation with powered exoskeletons has been investigated, with several studies demonstrating potential health benefits. For example, Stampacchia et al. (2016) found positive changes in activity limitations, symptoms, emotions, and overall quality of life [23]. Similarly, some case studies [37,47] reported an increase in another domain of health-related quality of life measures (physical functioning, physical role function, physical pain, general health, vitality, and physical well-being), which was confirmed at follow-up six months after rehabilitation. Considering that the results are based on small samples and the paucity of studies, further evidence is needed to understand these aspects better.

In summary, exoskeletons not only have the potential to help people with SCIs regain mobility but could also offer a number of secondary physical benefits that could reduce complications arising from prolonged immobilization in a wheelchair. In future studies, various training programs must overcome the perils of heterogeneous populations (injury severity and neurological level of SCIs) and methods (small sample sizes). Prolonged use of an exoskeleton at home or over a long period of time may prove these effective benefits.

## 4. The Exoskeleton in the Body: Exoskeleton Embodiment as an Upgrade to a Personalized Exoskeleton

**Figure 1:**
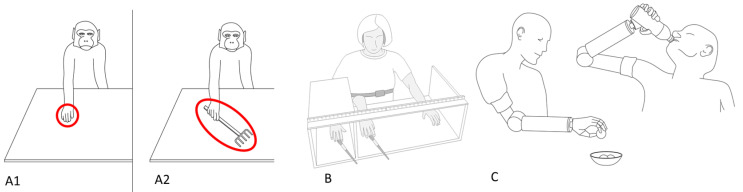
Embodiment and body ownership examples. (**A1**) Hand-related neuronal populations that normally react only to visual stimuli near the hand (**A2**) after repeated tool use and show similar activation patterns for the area surrounding the tool. (**B**) The classic rubber hand illusion show how artificial objects, using proper stimulation procedures, can be experienced as part of our own body. (**C**) The appropriate combination of training and features of prosthetic devices can lead to their integration into body representations, allowing for effective interaction with one’s body and environment.

Neuroscience and experimental psychology have shown that the brain can treat a tool as part of the body [48,49,50,51] (Figure 1).

Several studies have documented behavioral and neural changes following tool use. In blind people, the use of a long cane is perceived as an extension of the body’s senses, with the sense of touch being transferred from the hand to the endpoint of the assistive device [52]. In amputees, the use of a prosthesis is associated with the perception of effective arm lengthening [53]. Furthermore, brain imaging studies have shown cortical reorganization after the use of assistive devices [54,55,56], suggesting that external objects are incorporated into the neural representation of the body [57]. Body representation is thus plastic and appears to be able to include salient artificial tools [58,59] and even assistive devices [60].

The close link between the assistive device and body awareness and perception, referred to as embodiment, is the most crucial factor [48] influencing user acceptance, competence, and rejection [61]. The embodiment of a prosthetic aid can improve the competence and safety of movement, thereby reducing physical effort and injuries caused by the use of the tool [48,49] and offering new ways to restore interactions between the body and the world. However, the embodiment is a highly complex and plastic process [62] that operates on sensory–motor maps and requires interplay between the perception of afferent and efferent bodily signals and the cognitive evaluation of these states to influence brain experiences [48,49].

Despite the awareness that only bioinspired robots can improve rehabilitation [63], there have been very few attempts to develop person-centered wearable robotics and training to improve embodiment [64]. Adoption of the right assistive device depends on several factors [65], not only the design of the assistive device (comfort, availability, functionality, and durability) but also the needs and limits of the patient (physical and cognitive impairment and goals).

How can these devices be appropriately controlled by the brain as one’s real body part? In amputees, appropriate redirection of physiological sensations from a limb to the phantom limb map drives a perceptual shift towards the embodiment of the device [61]. Unable to use motor, somatic, and proprioceptive information, individuals with SCIs may experience constant pain that adversely affects their body awareness, perception, and predictions [66]. A reduction in the privileged sensory and proprioceptive information of the body results in a lack of body awareness and makes it difficult to modulate movement and tool use prediction to shape the mental simulation process [48,49].

Currently, brain–computer interfaces probably best represent the concept and promise of the embodiment of an exoskeleton. In particular, it is necessary to execute the control of movement via neural interfaces capable of recording cortical electrical activity and translating it into commands. In experiences such as the exceptional procedure observed by Benabid et al. [67], the most pertinent example of the personalization of an intervention was achieved in a mutual learning and adaptation process involving the patient, the devices, and a multidisciplinary clinical team. However, being very invasive and extremely expensive, this pathway is unsuitable for a large population and needs, especially of large and high-level multidisciplinary teams. Although successful in augmenting leg and arm motions, the use of exoskeletons still neglects the important role that in the transmission of signals could play in body awareness and sentience in patients with SCIs.

## 5. The Exoskeleton with the Body: Interaction between Different Signals and Homeostasis as the Next Level for Personalized Exoskeleton Rehabilitation

Considering the currently available information and the growing body of literature, it is easy to understand why promoting an adequate exoskeleton–patient interaction is onerous and controversial in terms of both appreciation and efficacy. One sparsely researched resource is interoception, which refers to a constant flow of bodily and visceral-derived data informing and regulating higher cognitive activities that rarely cross the threshold of awareness [68].

An approach focused on embodiment and, as a consequence, on a transparent unified experience of thought and action requires a full understanding of the potential impact of SCIs on the information that comes from the body. A curious but explanatory effect of this complexity is that spinal lesions do not apparently affect the sense of ownership for the supralesional part of the body but, rather, the stability of the body schema—namely, the representation of the body in the space necessary for action [69]. While touch and vision do play pivotal roles in ownership, proprioception is dominated by signals of internal origin. The fact that the lesion seemingly also alters the proprioceptive abilities for the body part not directly affected suggests that an approach oriented to the entire body experience is necessary to restore body awareness so that a profitable interaction with assistive devices can be supported.

Before a successful embodiment of prosthetic legs, information is needed on the systematic dysfunction of how the human brain in patients with spinal lesions adapts to changes in body signals [66]. Individuals with SCIs exhibit disturbances in their homeostatic balance [66,69,70]. The loss of somatic and visceral information leads to the misperception of interoceptive signals and the maintenance of homeostatic stability [66,69,70]. This contributes to the distortions of body awareness and internal conscious representation of the body.

Homeostatic stability requires a balance between internal feedback and environmental context. This facilitates the identification of correct interoceptive signals and allostatic interoceptive signals [71,72]. The integrity of the system is maintained by comparing interoceptive mechanisms and visceral inputs [71,72], allowing the former to be constantly updated in relation to the state of the body [73].

**Figure 2:**
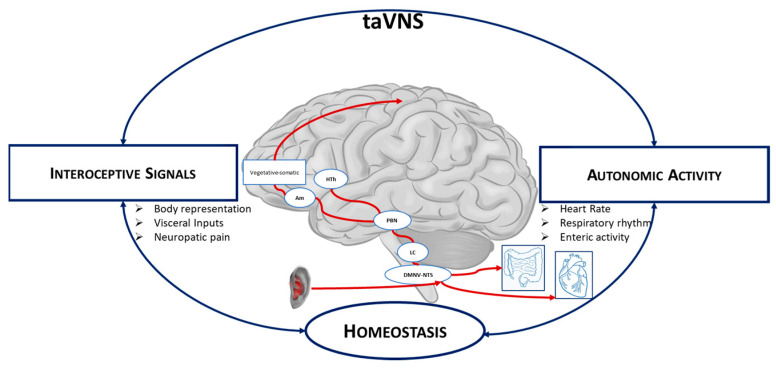
taVNS stimulation of the auricular branch of the vagus nerve (VN) projects to the nucleus tractus solitari (NTS), continuing to the locus coeruleus and parabrachial nucleus. From the parabrachial nucleus, it propagates to various subcortical and cortical brain regions. HTh: hypothalamus; PBN: parabrachial nucleus; LC: locus coeruleus; NTS: nucleus tractus solitary; DMNV: dorsal motor nucleus of the vagus nerve.

Allostatic interoceptive inferences are considered the first low-level processes to adapt to the demands of the external environment. The vagus nerve is a key homeostatic component of the parasympathetic branch of the autonomic nervous system (ANS) and carries ascending interoceptive sensory information via the internal organs and the enteric nervous system. Visceral and interoceptive signals, particularly respiratory and cardiac signals, reach the superior cerebral areas through the vagal system. One of the major interoceptive targets of the brainstem, the nucleus tractus solitarius (NTS), is fundamental for the control of physiological states and is connected mainly via the thalamus to several interoceptive areas, such as the somatosensory cortex and the insula [74]. These networks play an important role in body representation and the sense of body ownership. Accordingly, the modulation of autonomic signals can affect interoceptive signals. Therefore, noninvasive vagal stimulation (taVNS), a technique currently used in the treatment of various diseases [75], can presumably be used to modulate interoceptive signals [76] and, consequently, enhance body awareness by strengthening the connection between different body parts and the overall body representation (Figure 2). In fact, some studies reported that the synchronization of taVNS with the respiratory rhythm caused a stronger activation of some vagal targets, such as the NTS or neuromodulating nuclei (i.e., locus coeruleus and raphe nuclei), synchronizing the stimulation with the expiratory phase [77,78].

Interestingly, the vagal pathway is not involved in spinal lesions. For this reason, taVNS offers several advantages for people with SCIs [79]. To consider an assistive device as part of one’s body, two elements are essential for people with SCIs: the use of an assistive device capable of partially restoring the lost functionality (i.e., movement) and the need to experience better body awareness, especially internal body awareness [80,81]. In this sense, the use of taVNS during rehabilitation training with assistive devices (e.g., an exoskeleton) is likely to allow subjects, especially those with more severe injuries, to feel like they have an “insentient” body again.

Improving autonomic signals and, consequently, interoceptive signals could also restore homeostasis in people with SCIs. Moreover, coordination between respiratory rhythm and movement could be another approach for rehabilitation with an assistive device, especially an exoskeleton [82]. Adaptation to the voluntary breathing rhythm could allow synchronization of the subject with the exoskeleton’s movement to improve their ability to walk autonomously. In addition, the analgesic effects of taVNS could also be used to treat neuropathic pain, which could contribute to less change in body awareness and further improve embodiment effects [79]. With this finding, the interaction between different signals (i.e., interoceptive and autonomic) and the maintenance of homeostasis could be considered the next stage of “personalized” exoskeleton rehabilitation.

## 6. Conclusions

Despite the technological advancements witnessed over the past few decades, only limited success has been reported in the usage of exoskeletons [4,13]. Both clinicians and engineers have recognized the possibility that people with SCIs could reject their bionic legs, because they do not yet feel like biological ones [48,49,80,83]. However, little attention has been paid to how the human body responds to and supports such technological innovations in terms of active body control and sensing [48,49,80,83,84]. These concerns are particularly relevant for patients with body paralysis and numbness due to SCIs. In this sense, it would be the most effective and useful to focus on personalized interventions that can emphasize residual abilities after SCIs. Some useful signals seem to be the autonomic and non-perceptual ones, which, when combined, can provide the patient with a useful aid in general rehabilitation and the acceptance and use of the exoskeleton.

## Figures and Tables

**Table 1:** Robotic exoskeletons approved for rehabilitation by the Food and Drug Administration.

Device	Approved for Use	Injury Level
ReWalk™ (ReWalk Robotics Inc., Marlboro, MA, USA and Yokneam, Israel)	Rehabilitation and personal mobility (community)	T4–L5 (Rehabilitation) T7–L5 (Personal Use)
Indego™ (Parker Hannifin Corporation, Cleveland, OH, USA)	Rehabilitation and personal mobility (community)	T4–L5 (Rehabilitation) T7–L5 (Personal Use)
Ekso^®^ (Ekso Bionics, Berkley, CA, USA)	Rehabilitation	T4–L5 AIS A–D C7–T3 if AIS D

## Data Availability

Not applicable.
